# Multiscale landscape genetic analysis identifies major waterways as a barrier to dispersal of feral pigs in north Queensland, Australia

**DOI:** 10.1002/ece3.10575

**Published:** 2023-09-28

**Authors:** James Ryan, Peter J. Prentis, Susan Fuller

**Affiliations:** ^1^ School of Biology and Environmental Science Queensland University of Technology Brisbane Queensland Australia; ^2^ Centre for Agriculture and the Bioeconomy Queensland University of Technology Brisbane Queensland Australia

**Keywords:** invasive species, isolation by resistance, landscape genetics, microsatellite DNA markers, pest management, *Sus scrofa*

## Abstract

Feral pigs (*Sus scrofa*) are a destructive and widespread invasive pest in Australia. An understanding of feral pig movement is required to develop management strategies to control feral pigs in Australia. Because landscape structure can have a strong influence on animal movement, it is important to determine how landscape features facilitate or impede the movement of feral pigs. Consequently, we conducted a landscape genetic analysis of feral pig populations in the Herbert region of far north Queensland, Australia, to determine management units and provide recommendations to better inform feral pig population control strategies. Using microsatellite data obtained from 256 feral pig samples from 44 sites, we examined feral pig population structure at multiple spatial scales for univariate and multivariate landscape resistance surfaces to determine the optimal spatial scale and to identify which of the nine landscape features tested impede or facilitate feral pig gene flow. Only weak genetic structure was found among the 44 sampling sites, but major waterways were identified as a minor barrier to gene flow, and an isolation by distance model was supported. We also found that highways facilitated gene flow across the study area, and this suggests that they may act as movement corridors or indicate translocation of feral pigs. Additionally, incorporating a second spatial scale enhanced the ability of our landscape genetics analysis to detect the influence of landscape structure on gene flow. We identified three management units based on natural barriers to gene flow and future targeted control should be undertaken in these management units to deliver sustained reduction of feral pig populations in the Herbert region. This study demonstrates how a landscape genetic approach can be used to gain insight into the ecology of an invasive pest species and be used to develop population control strategies which utilise natural barriers to movement.

## Graphical abstract

We conducted a multiscale landscape genetics analysis of feral pigs in far north Queensland, Australia. Waterways were identified as a barrier, while highways facilitated gene flow. Three management units were identified and will inform future targeted control strategies.

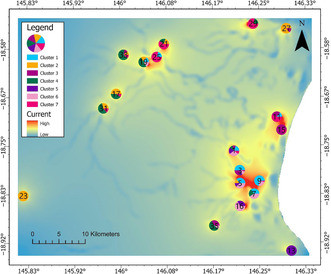

## 1 INTRODUCTION

One of the most destructive invasive species in Australia is *Sus scrofa*, the feral pig. Feral pigs are estimated to cost agricultural industries in Australia up to $100 million per annum (Choquenot et al., 1996; McLeod, 2004). In some cases, crops are a heavily exploited food resource (Gentle et al., 2015; Wishart et al., 2015; Wurster et al., 2012), but feral pigs also prey on lambs (*Ovis aries*) (Choquenot et al., 1997; Pavlov et al., 1981; Plant et al., 1978; Wishart et al., 2015) and consume native species including Lord Howe Island woodhen (Tricholimnas sylvestris; Miller & Mullette, 1985), snake‐necked turtles (*Chelodina rugosa*; Fordham et al., 2006), and the eggs of flatback sea turtle (*Natator depressus*; Whytlaw et al., 2013), olive ridley sea turtle (*Lepidochelys olivacea*; Whytlaw et al., 2013), and hawksbill sea turtle (*Eretemochelys imbricata*; Whytlaw et al., 2013). Additionally, feral pigs are a host of various pathogens which cause human illnesses such as hand, foot and mouth disease (Doran & Laffan, 2005; Pech & Hone, 1988), leptospirosis (Mason et al., 1998) and *Salmonella* infection (Ward et al., 2013). Feral pigs are widespread in Australia, but population management has been largely ad hoc with operational boundaries defined according to geography, government jurisdiction and landholder agreements (Choquenot et al., 1996). This strategy has led to inefficient management strategies resulting in only short‐term population decline where feral pigs from the same subpopulation reinvade because the population extends further than the management boundary (Cowled et al., 2006). When population boundaries are unknown, molecular data can be used to estimate population structure and define management units for effective population reduction (Manel et al., 2003; Moritz, 1994).

Understanding the influence of landscape features on gene flow in invasive species is important for developing effective management strategies and identifying potential management units. Landscape genetics is a useful tool which allows the identification of landscape features that are associated with observed patterns of population structure (Manel et al., 2003). Furthermore, the combination of geographic information systems (GIS) and molecular data allow landscape genetic models to be assessed over multiple spatial scales, enhancing the capacity of landscape genetic models to identify landscape features that promote or reduce gene flow in a given species (Cushman et al., 2016; Krishnamurthy et al., 2016; Winiarski et al., 2020; Zeller et al., 2014, 2017). Consequently, using a landscape genetic approach to define invasive species population boundaries can result in enhanced targeted control of the pest species by limiting connectivity between management units (Zalewski et al., 2009).

Studies have shown that wild pig population structure often conforms to isolation by distance (IBD) pattern (e.g. Choi et al., 2014; Cowled et al., 2008; Frantz et al., 2009, 2012; Renner et al., 2016; Rutten et al., 2019) which means there are fewer migrants shared by feral pig populations as distance between populations increases. Australian and international studies that have reported isolation by distance have found that distinct subpopulations exist within a single study area (Delgado‐Acevedo et al., 2021; Lopez et al., 2014; Spencer & Hampton, 2005), and it is possible that landscape features may be limiting gene flow between subpopulations. For example, Lopez et al. (2014) found three distinct subpopulations of feral pigs in far‐north Queensland. Isolation by distance was detected within these subpopulations over a spatial scale of between 25 and 35 km, however, no IBD was detected among subpopulations. Spatial landscape analysis was not undertaken and therefore environmental features associated with management unit boundaries could not be identified. A study from south‐west Western Australia found genetic structuring across river systems, but not along a river catchment, indicating feral pigs are utilising rivers as movement pathways (Hampton et al., 2004). Similarly, Cowled et al. (2008) found that feral pigs moved along rivers in low rainfall environments. Notably, several Australian (Hampton et al., 2004; Spencer & Hampton, 2005) and international studies (Delgado‐Acevedo et al., 2021; Hernández et al., 2018; McCann et al., 2014, 2018; Nikolov et al., 2009; Scandura et al., 2011; Tabak et al., 2017; Vernesi et al., 2003) have identified the possibility of illegal translocation which may be linked to transport routes (such as highways and roads; Medley et al., 2015; Spencer & Hampton, 2005). The impact of waterways and transport routes on feral pig gene flow in high rainfall environments has not been formally tested using landscape genetics modelling and is needed to determine management unit boundaries.

The current study aimed to provide the first landscape genetic analysis, using spatial mapping and statistical modelling, of feral pigs in far north Queensland. Given the feral pig population density in the lowlands of the Wet Tropics of Queensland is estimated to be 3.1 pigs per km^2^ (Mitchell, 2003) and the associated economic cost and environmental impact, targeted, effective and sustained control is imperative, but can only be achieved through a comprehensive landscape genetic analysis of feral pig population structure. In the current study, we considered both isolation by distance and isolation by resistance (McRae, 2006) models in our analysis of population genetic structure. We also used multiple spatial scales for univariate and multivariate landscape resistance surfaces (RSs) to determine the optimal spatial scale to examine feral pig population structure and identify landscape features that impede or facilitate feral pig gene flow. Our results will enable local governments, communities and feral pig management groups to make informed, effective and targeted feral pig control decisions based on the identification of management units and natural landscape barriers.

## 2 METHODS

### 2.1 Sampling

**FIGURE 1:**
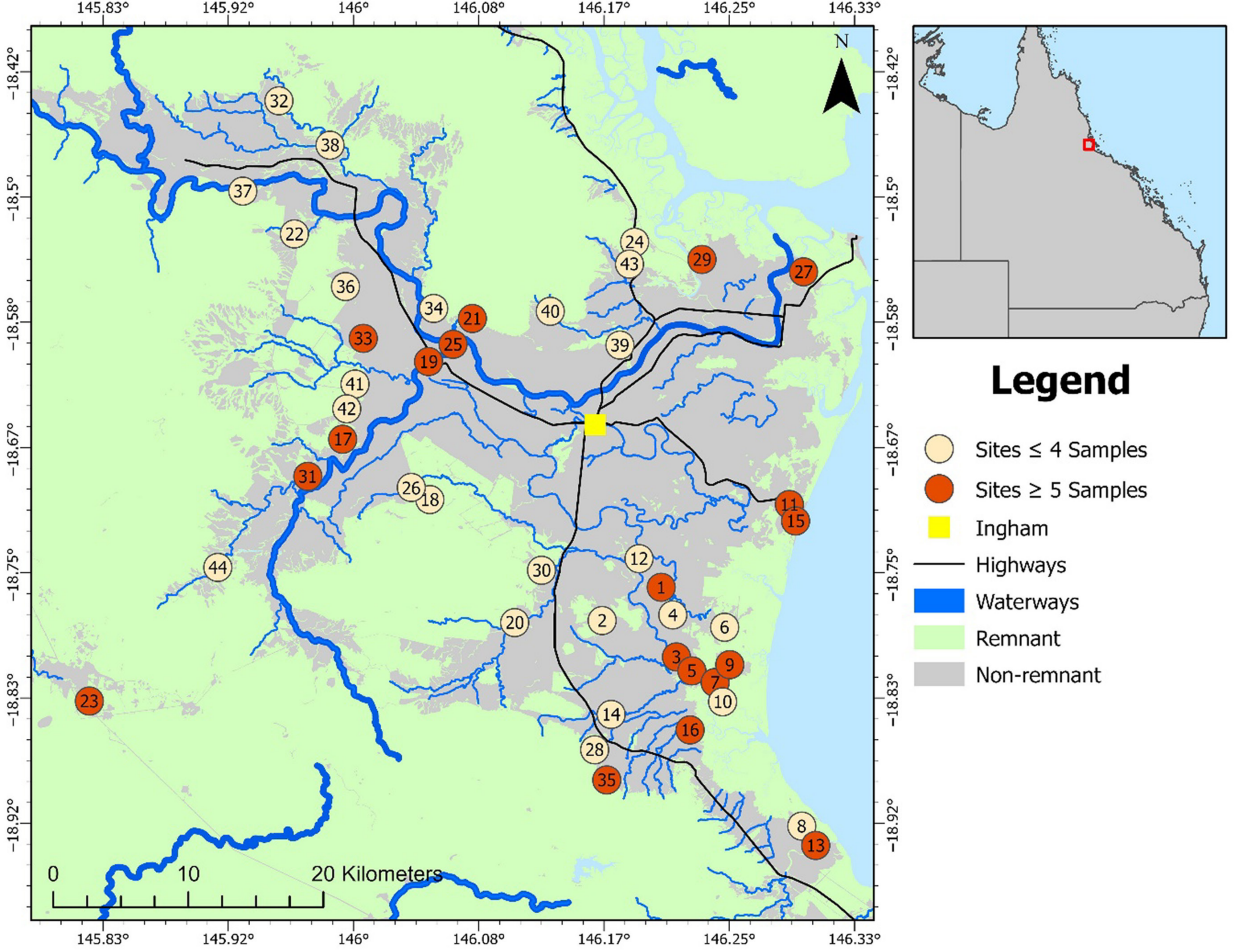
Map of the study area in far‐north Queensland Australia including feral pig (*Sus scrofa*) sampling sites (numbered 1–44), highways, waterways, remnant and non‐remnant vegetation and the major urban centre in the region (Ingham). Remnant vegetation is defined as vegetation that has not been cleared or where vegetation has been partially cleared but the vegetation that remains has greater than 70% of the relative height and occupies greater than 50% of the relative canopy cover to the undisturbed vegetation height and cover (Queensland Government, 2023). Non‐remnant is the absence of remnant vegetation.

The Herbert region has been largely cleared of remnant vegetation at low elevations, with most remaining remnant vegetation patches confined to coastal areas, river systems and elevated areas (Figure 1). These cleared lowland areas are now used primarily for agricultural production, mainly sugarcane and cattle grazing areas. The Bruce Highway is the major north/south highway on the east coast of Australia and runs through the study area converging with a secondary main road which runs east/west through the town of Ingham, the largest urban centre in the study area (with a population of 4767; Australian Bureau of Statistics, 2011). The Herbert region has many ephemeral and perennial waterways, the largest of which is the Herbert River with a width of >1 km at its widest point. The region experiences an average annual rainfall in excess of 2000 mm (Bureau of Meteorology, 2021).

Microsatellite data for 385 feral pigs at eight unlinked loci (SW240, SW632, SW857, SW911, SW936, SW951, S0002 and S0068; Alexander et al., 1996) were provided from a previous study (Di Bella et al., 2014). Sampling was undertaken in 2012 and 2013 by authorised and licenced contractors. Feral pig tissue samples were collected through various methods (trapping, dogging and baiting; Di Bella et al., 2014). Ear and tail tissue were provided by Herbert Cane Productivity Services Ltd and Hinchinbrook Community Feral Pig Management Program (Animal Ethics Tissue Use Notification #1300000039). Each feral pig was allocated to a weight class (0–5, 5–10, 10–40, 40–60, 60–80, 80–100 and 100–150 kg). We filtered the data by weight class and removed pigs that were 10 kg or less in weight from our analysis. This was to limit the inclusion of related pigs, particularly young juveniles, in the genetic analysis. We acknowledge that this approach of omitting piglets from the analysis does not completely eliminate the possibility of including related individuals, particularly because the sampling methods employed may trap feral pigs moving in a sounder (i.e. more likely to be related). After excluding feral pigs based on weight, a total of 256 feral pigs sampled from 44 sites (located >1 km to 66.5 km apart throughout the Herbert region) remained and were used for genetic analysis (see Figure 1 for site locations).

### 2.2 Genetic diversity and structure

We calculated standard genetic diversity measures for sites with five or more samples. We used Arlequin v3.5.2.2 (Excoffier & Lischer, 2010) to calculate inbreeding coefficients (*F*_IS_; Weir & Cockerham, 1984) and linkage disequilibrium (Lewontin & Kojima, 1960; Slatkin, 1994; Slatkin & Excoffier, 1996) using 10,000 permutations each, and deviations from Hardy–Weinberg equilibrium (Guo & Thompson, 1992; Levene, 1949) using a burnin period of 100,000 steps and Markov chain of 1000,000 steps. To adjust significance following multiple comparisons, we applied a Bonferroni correction (Rice, 1989) to linkage disequilibrium and Hardy–Weinberg equilibrium tests. To calculate the mean number of alleles (*N*_A_), expected heterozygosity (*H*_E_) and observed heterozygosity (*H*_O_) we used the R package adegenet v2.1.3 (Jombart, 2008) and to calculate allelic richness (*A*_R_; El Mousadik & Petit, 1996; Hurlbert, 1971) we used the R package PopGenReport (Adamack & Gruber, 2014).

We used Arlequin v3.5.2.2 to calculate pairwise linearised F_ST_ (Slatkin, 1995) using 100 permutations, and we corrected the significance for multiple comparisons (Rice, 1989). To assess the relationship between linearised pairwise F_ST_ and geographic distance we performed a Mantel test (Mantel, 1967) using the R package ade4 v1.7–16 (Dray & Dufour, 2007). Statistical significance was determined based on 10,000 permutations. We used a Discriminant Analysis of Principal Components (DAPC; Jombart et al., 2010) to determine the number of genetic clusters (*K*) using the R package adegenet v2.1.3. DAPC maximises the differentiation between predefined clusters whilst minimising differentiation within groups, and unlike other model‐based approaches, DAPC relies on no underlying Hardy–Weinberg equilibrium or linkage disequilibrium assumptions (Jombart et al., 2010). There are two stages during the DAPC; first, genetic data fitted to predefined genetic clusters are transformed via principal component analysis (PCA); second, the principal components (PCs) of the PCA are then transformed with a linear discriminant analysis (LDA). To define clusters, *K* values were assessed from *K* = 1 to *K* = 20 using the *K*‐means clustering algorithm *find. clusters()* in adegenet. For this step, all 68 PCs were retained (Jombart & Collins, 2015). We determined the optimal value for *K* based on Bayesian information criterion (BIC). DAPC can benefit from using fewer PCs than the *K*‐means clustering algorithm to avoid overfitting during discrimination. To define the optimal number of PCs to retain during DAPC, we used the *xvalDpac()* function. We used the *scatter()* function to visualise the degree of genetic structuring between clusters. In addition, we used Bayesian methods implemented in STRUCTURE v2.3.4 (Falush et al., 2003; Pritchard et al., 2000) to independently determine the number of genetic clusters. We used 20 runs to evaluate *K* from 1 to 15, with a burn‐in fraction of 100,000, and 100,000 Markov Chain Monte Carlo (MCMC) repetitions using the admixture and correlated allele frequencies model. We determined the optimal value for *K* based on the STRUCTURE analysis using the ∆*K* method (Evanno et al., 2005) and the Puechmaille method (Puechmaille, 2016) as implemented in the online software StructureSelector (Li & Liu, 2018). Briefly, the Puechmaille method uses the median of medians (MedMedK*)*, the median of means (MedMeanK), the maximum of medians (MaxMedK) and the maximum of means (MaxMeanK) to identify the optimal value for *K*. To visualise the optimal values for *K*, we used Clumpak (Kopelman et al., 2015). To visualise genetic dissimilarity among sites, a PCA was performed in the R package ade4 v1.7‐16 retaining all PCs for the analysis.

### 2.3 Developing resistance surfaces

We developed nine resistance surfaces based on landscape features that we hypothesised would impede or facilitate gene flow in feral pigs. We cropped layers to a 1 km buffer beyond the furthest sampling site to the north, south and west, while the eastern extent was the Australian coastline of the sampling region in far‐north Queensland. We added this 1 km buffer to the outside edge of our study area to minimise potentially biased current density at the edges of our resistance surfaces (Koen et al., 2010). This avoids isolation by resistance modelling including biologically unreasonable dispersal pathways in resistance surface optimisation (Cameron et al., 2019).

Of the nine resistance surfaces, five were categorical binary layers representing the presence/absence of landscape features for a variety of land use classes. These layers were developed by rasterising vector data retrieved from Queensland Spatial Catalogue – QSpatial (http://qldspatial.information.qld.gov.au/catalogue/custom/index.page; see Appendix S1 for original layer names). Each raster was developed at a cell size of 100 m. The remaining four layers were continuous resistance surfaces representing elevation, slope, topographic wetness index (TWI; retrieved from CSIRO; https://data.csiro.au/dap/home?execution=e2s1) and foliage projective cover (FPC; a measure of the percentage of ground area occupied by woody vegetation based on data collected via remote sensing; retrieved from Qspatial; https://qldspatial.information.qld.gov.au/catalogue/custom/index.page). Essentially, this spatial layer is a measurement of vegetation density and is an alternative way of incorporating remnant vegetation into the landscape genetics analysis. Elevation and FPC were resampled from a cell size of 25–100 m and TWI from 101 to 100 m using the Resample (Data Management) tool in Arcmap v10.6 and the bi‐linear resampling technique. To generate the slope layer we used the Slope (Spatial Analyst) tool in Arcmap v10.6 using the 25 m resolution elevation raster retrieved from Qspatial as the input feature. We employed the geodesic method and set output measurement to degrees. Then we resampled slope from 25 to 100 m using the bi‐linear resampling technique.

To evaluate layers at a second spatial scale, we used a method similar to that of Winiarski et al. (2020). Briefly, a Gaussian kernel smoothing at 1000 m was applied to each spatial layer using the R package gridkernel (https://github.com/ethanplunkett/gridkernel). A Gaussian kernel applies a normal distribution to weight highest the cells which are closest to the focal cell and has been reported to incorporate spatial scale more realistically than alternative approaches (Winiarski et al., 2020). The Gaussian kernel smoothing process ‘smooths’ over features in each RS which are smaller in area than the smoothing bandwidth, i.e. features less than 1000 m in width in any direction. This allows isolation by resistance modelling to apply different resistances to a feature within a RS depending on its size, for example, a large water body may be assigned a higher resistance to gene flow than a smaller body of water. The kernel smoothing process also transforms each categorical binary surface into a continuous surface. This resulted in 18 landscape‐derived resistance surfaces for the landscape genetics analysis.

### 2.4 Landscape genetics resistance surface optimisation

We used the R package ResistanceGA v4.0‐14 (Peterman, 2018; https://github.com/wpeterman/ResistanceGA) to determine which landscape feature or combination of landscape features best explained the observed genetic structure. This package uses a genetic algorithm (R package GA; Scrucca, 2013) to adaptively explore and maximise the relationship between matrices of pairwise resistance distance and genetic distance. This is done using a linear mixed effects model with maximum likelihood population effects (MLPE; Clarke et al., 2002), implemented using the R package LME4 (Bates et al., 2015). This statistical framework accounts for non‐independence among pairwise data (Clarke et al., 2002; Van Strien et al., 2012) and has been shown to perform better than alternative modelling techniques used in other landscape genetics analyses (Peterman et al., 2019; Shirk et al., 2017).

First, we performed single surface optimisation for each of the 18 resistance surfaces using pairwise linearised F_ST_ as the response variable and pairwise resistance distances calculated in CIRCUITSCAPE v4.0.5 (McRae et al., 2013) using an eight‐neighbour connection scheme. In addition to the 18 resistance surfaces, ResistanceGA incorporates two alternative models: a distance model to test for isolation by distance (IBD), where all cells in the raster are set to a resistance of 1, and an intercept‐only null model without any landscape structure (i.e. island model). We used ResistanceGA function *SS_optim()* to optimise each individual resistance surface with the Akaike Information Criterion (AIC; Akaike, 1998) set to the objective criterion that the genetic algorithm worked to minimise during optimisation. We ranked model performance based on AIC corrected for finite sample size (AICc) according to the difference from the top‐ranked model.

Secondly, we performed multivariate optimisation to explore whether a range of landscape features better explained the observed feral pig genetic structure than the univariate models. To assess multivariate models, the three best‐supported models from the univariate analysis were modelled with every other surface at their best‐supported spatial scale. The Band Collection Statistics (Spatial Analyst) geoprocessing tool in ArcMap v10.6 was employed to assess raster combinations for correlation and to exclude any combination of resistance surfaces with a correlation >0.7 (Pearson's correlation coefficient) to avoid multicollinearity. We used the *MS_optim()* function to conduct the multivariate analysis, using the same model parameters as used in the univariate analysis. We also used the *Resist.boot()* function to conduct a 1000 bootstrap analysis subsampling 75% of sampling sites and refitting the MLPE model for each optimised resistance surface from both the univariate and multivariate analysis. We calculated the Akaike weight (ω_i_; relative likelihood of a model) and the number of times that a model was the best supported for each bootstrap iteration. We omitted several models from the bootstrapping analysis due to ResistanceGA converging on a resistance surface of functional equivalence to the distance model.

## 3 RESULTS

Tests for linkage disequilibrium were conducted for each of the eight loci across all 44 sites. Following Bonferroni correction (*α* < 0.00009), one comparison out of 532 pair‐wise tests was significant, subsequently, all loci were retained for further analysis. Hardy–Weinberg equilibrium analysis for sites with at least five samples revealed that after Bonferroni correction (152 comparisons; *α* < 0.0003), one locus at one site deviated from Hardy–Weinberg equilibrium with heterozygotic deficiency, as a result, all loci were retained in the subsequent analyses. Observed heterozygosity (*H*_O_) values ranged from 0.456 to 0.893 (Table 1), and inbreeding coefficient (*F*_IS_) values ranged from −0.325 to 0.257 (Table 1).

**TABLE 1:** Site‐specific (*N* ≥ 5) genetic diversity measures for feral pigs (*Sus scrofa*) in far‐north Queensland, Australia; number of samples (*N*), expected heterozygosity (*H*_E_), observed heterozygosity (*H*_O_), inbreeding coefficient (*F*_IS_), number of recorded alleles across 8 loci (*N*_A_), mean allelic richness (*A*_R_).

Site	*N*	*H*_E_	*H*_O_	*F*_IS_	*N*_A_	*A*_R_
1	10	0.586	0.458	0.257	38	2.826
3	49	0.614	0.589	0.011	49	2.968
5	8	0.615	0.574	0.116	37	2.988
7	17	0.613	0.623	0.004	41	2.933
9	26	0.550	0.515	0.021	44	2.742
11	7	0.566	0.661	−0.091	29	2.689
13	10	0.470	0.538	−0.092	21	2.198
15	7	0.580	0.696	−0.125	29	2.754
16	6	0.578	0.667	−0.063	29	2.722
17	10	0.581	0.544	0.098	31	2.755
19	6	0.593	0.646	−0.014	30	2.822
21	5	0.635	0.600	0.165	33	3.062
23	7	0.642	0.893	−0.325	29	2.902
25	5	0.425	0.510	−0.295	19	2.142
27	7	0.540	0.625	−0.083	25	2.521
29	5	0.406	0.456	−0.153	18	2.012
31	7	0.585	0.580	0.058	32	2.810
33	9	0.610	0.653	−0.012	40	2.994
35	9	0.625	0.653	−0.029	37	3.014

### 3.1 Population structure

**FIGURE 2:**
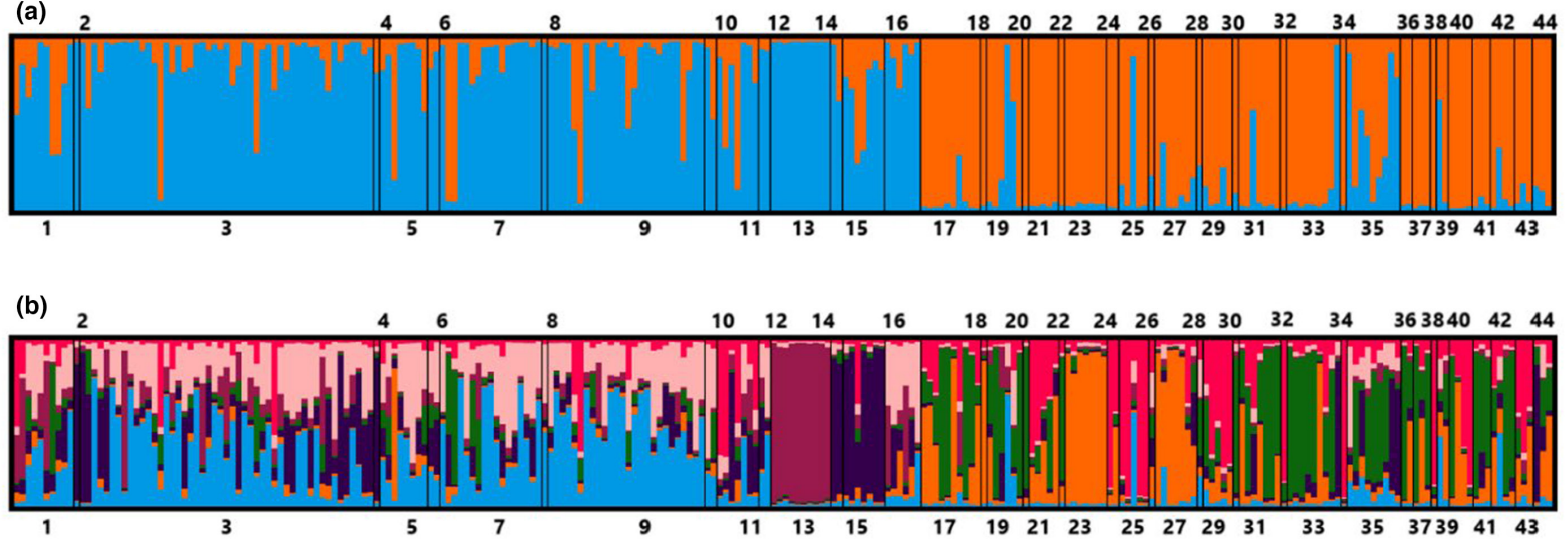
Bar plots of feral pig (*Sus scrofa*) in far‐north Queensland, Australia, from the STRUCTURE results for the optimal value of *K* determined by (a) The Evanno method of ∆*K* and (b) the Puechmaille method of MedMedK and MedMeanK.

The Mantel test revealed a significant correlation between genetic distance and geographic distance (*r* = 0.314, *p* = .034) indicating feral pigs in the study area exhibit a weak but significant isolation by distance pattern. The optimal value for *K* in the STRUCTURE analysis was identified as *K* = 2 using the Evanno method (Appendix S2) and using the Puechmaille method the optimal value was determined to be *K* = 7 using MedMedK and MedMeanK (Figure 2) or *K* = 8 using MaxMedK and MaxMeanK (Appendix S3).

**FIGURE 3:**
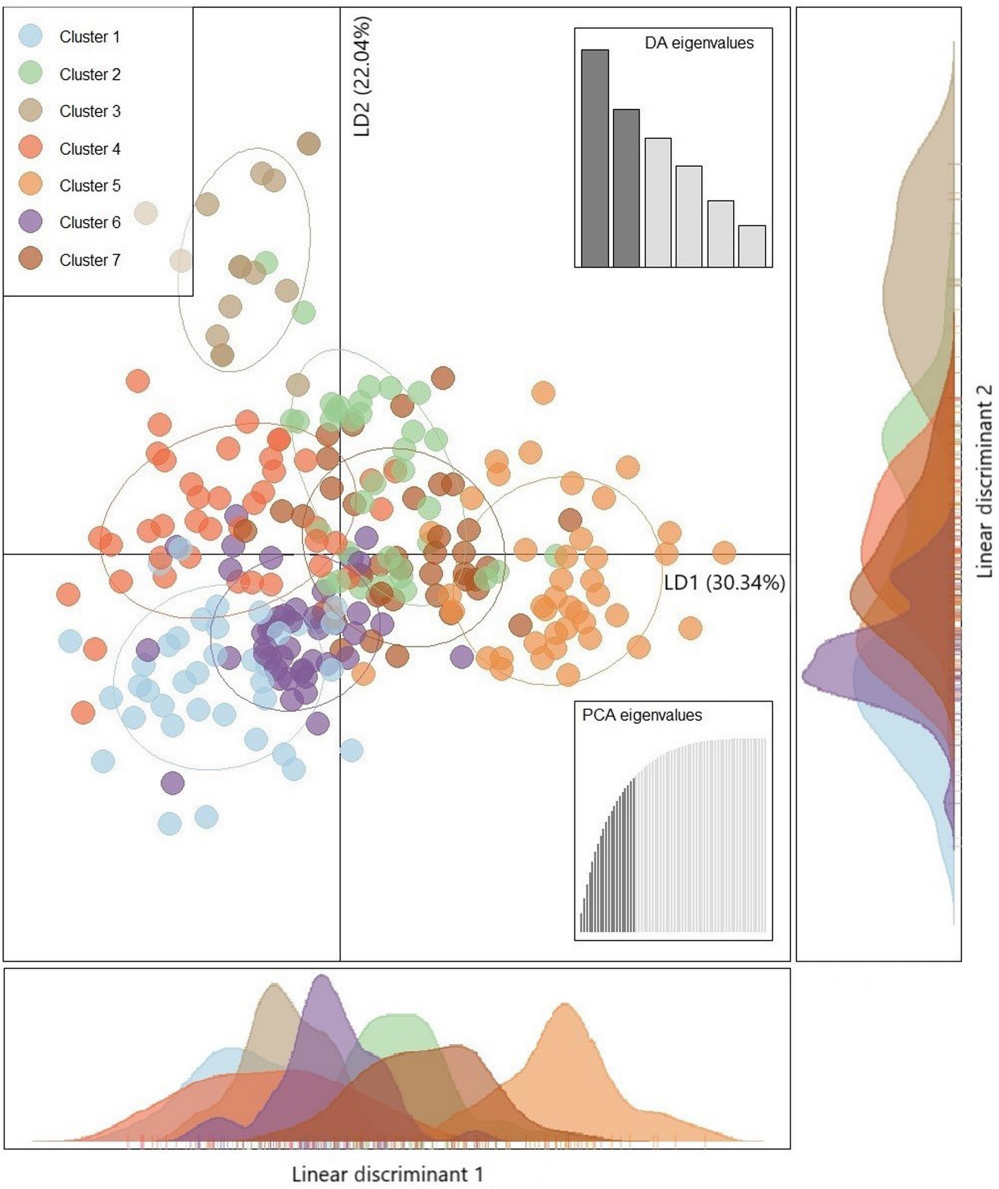
Ordination plot of Discriminant Analysis of Principal Components (DAPC) using all 256 feral pigs (*Sus scrofa*) across 44 sites in far‐north Queensland, Australia for *K* = 7. Retaining 20 Principal Components (PCs; 79.2% variation) and all six Linear Discriminants (LD).

The DAPC analysis revealed that the Bayesian Information Criterion (BIC) simulated values plateaued at *K* = 7 (Appendix S4). Cross‐validation identified 20 principal components (PCs; Appendix S5), accounting for 79.2% of variation, as the optimum number of PCs to retain during DAPC for the successful assignment of individuals into the correct cluster. The first linear discriminant (LD1) accounted for 30.34% of genetic variance between clusters, and the second linear discriminant (LD2) accounted for 22.04% of genetic variance between clusters (Figure 3). The ordination plot shows considerable genetic overlap between clusters 1, 2, 4, 6 and 7 with each cluster intersecting along both the LD1 axis and LD2 axis. Clusters 3 and 5 appear more distinct with separation along LD1 and LD2, respectively (Figure 3).

**FIGURE 4:**
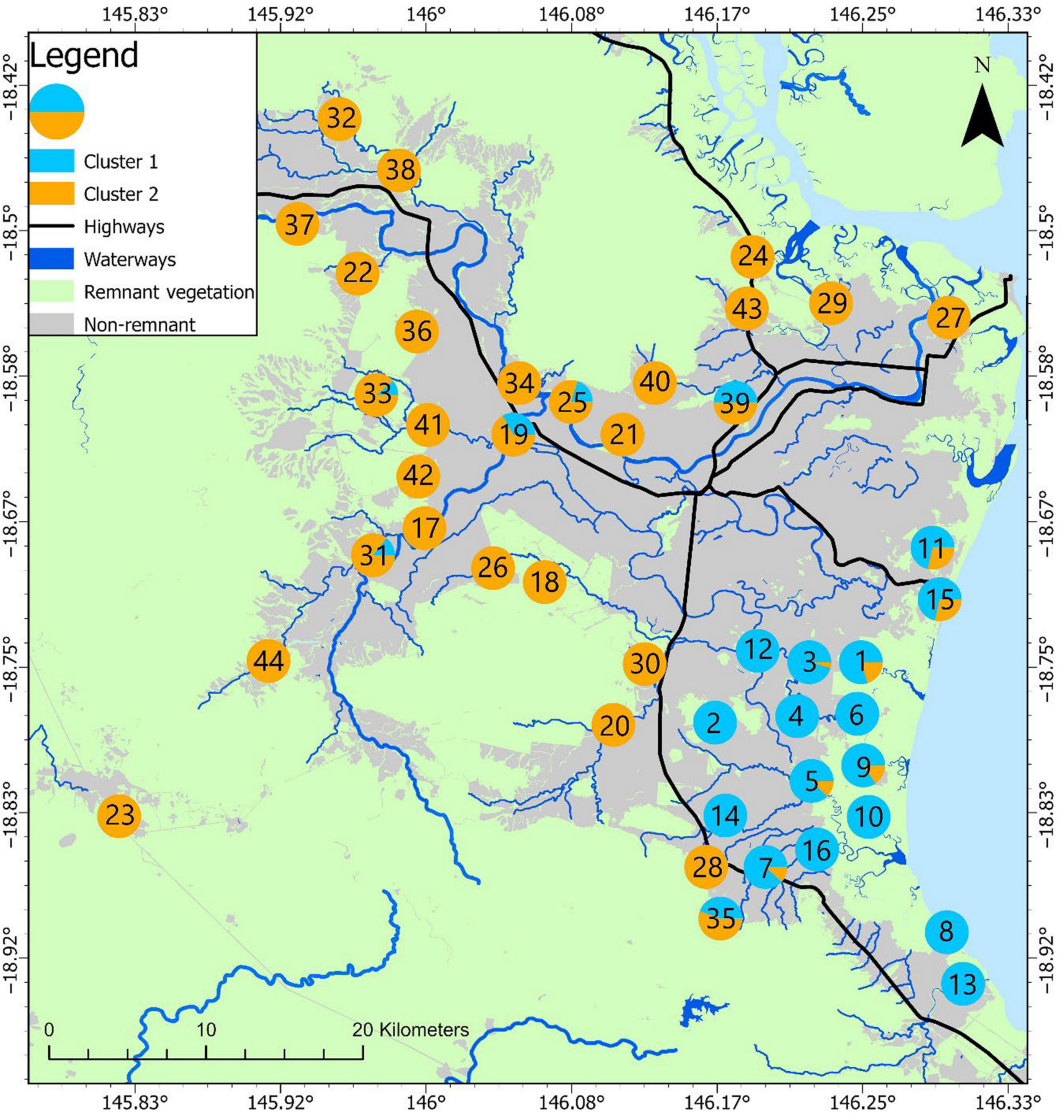
Proportion of feral pigs (*Sus scrofa*) in far‐north Queensland, Australia at each site assigned to each of the *K* = 2 clusters determined using the Evanno method (∆*K*) during the STRUCTURE analysis.

**FIGURE 5:**
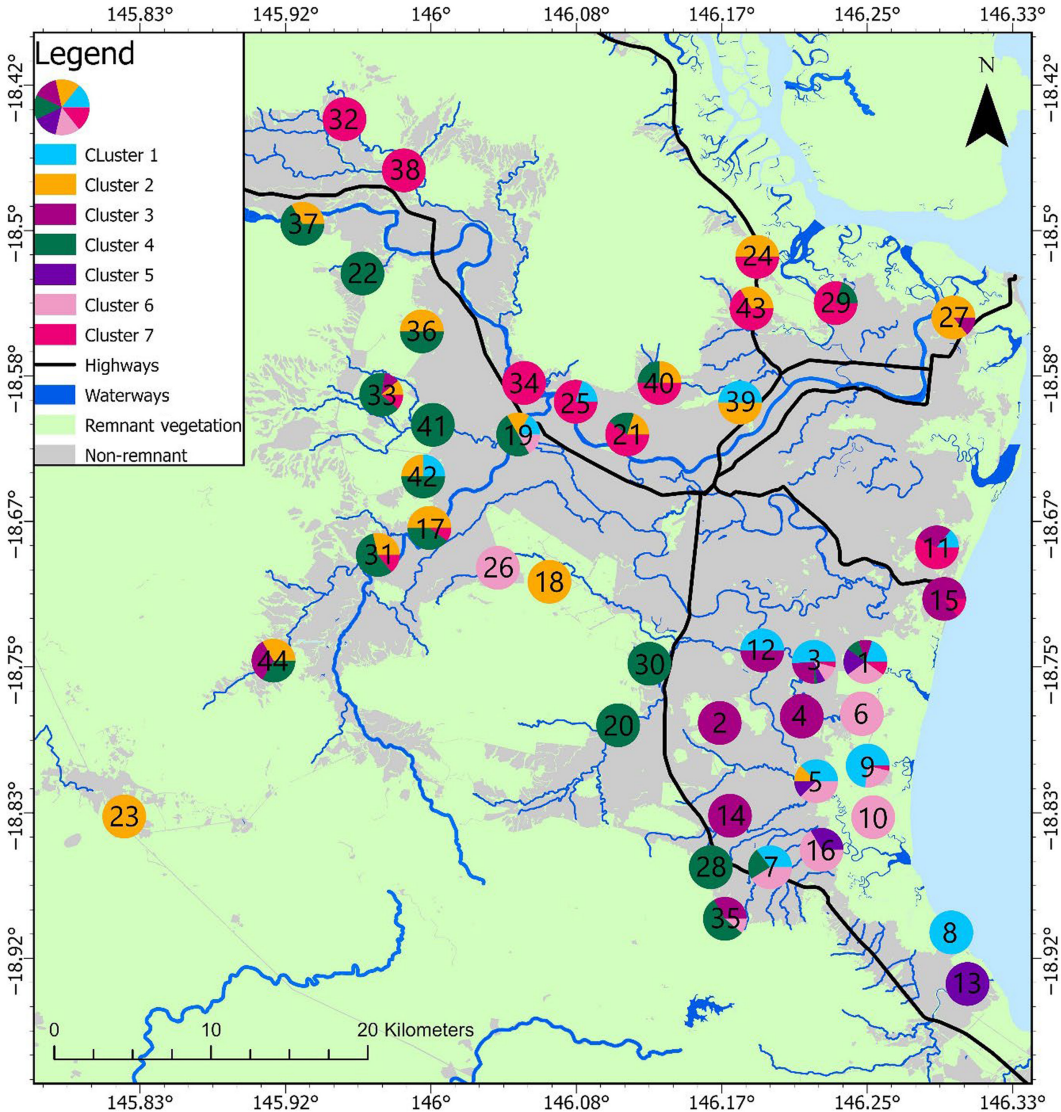
Proportion of feral pigs (*Sus scrofa*) in far‐north Queensland, Australia at each site assigned to each of the *K* = 7 clusters determined using the Puechmaille method (MedMedK & MedMeanK) during the STRUCTURE analysis.

Some geographic partitioning among clusters can be seen in both the *K* = 2 and *K* = 7 scenarios (Figures 4 and 5). At *K =* 2, sites which are predominantly comprised of individuals assigned to cluster 1 are exclusively found in the south of the study area, and most sites predominantly comprised of individuals from cluster 2 are found in the north of the study area, with a few exceptions in the south of the study area. At *K* = 7, individuals from clusters 1, 3, 5 and 6 are predominantly found in the south of the study area. Cluster 7 is predominantly represented in the north/north‐east of the study area and can visually be discerned as north of the Herbert River. Cluster 4 is predominantly represented in the north‐west/west of the study area, west of the Herbert River however, there are also a few sites in the south along the primary highway which are also predominantly cluster 4.

**FIGURE 6:**
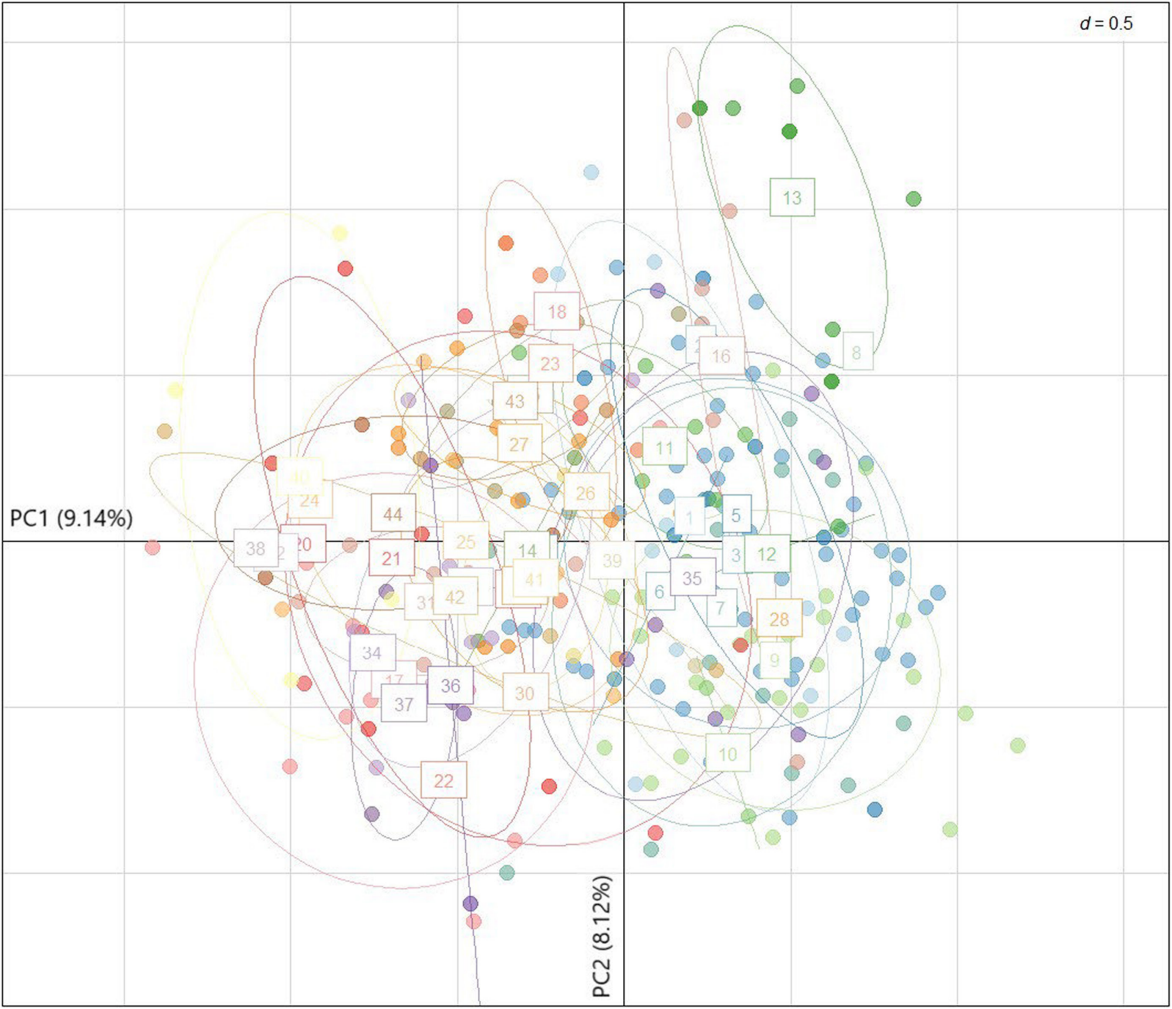
Principal component analysis (PCA) ordination plot of feral pigs (*Sus scrofa*) in far‐north Queensland, Australia, grouped by site. All Principal Components (PCs) were retained. Principal Component 1 (PC1) accounted for 9.14% of genetic variance and Principal Component 2 (PC2) accounted for 8.12% of genetic variance.

Principal component analysis did not reveal any strong clustering of individuals within sites or among geographically proximate sites (Figure 6). Along PC1, which accounted for 9.14% of genetic variance, there is a cline with northern sites predominantly plotted to the left of the origin, and southern sites plotted to the right. PC2 accounted for 8.21% of genetic variance, but there is no pattern in the ordination plot with any obvious relation to geography. Noteworthy is the differentiation of site 13. Linearised pairwise F_ST_ values for site 13 range from 0.200 to 0.606 (Appendix S6), including only one non‐significant pairwise comparison after Bonferroni correction (pairwise comparison with site 25; linearised F_ST_ = 0.522, *p* = .009; Bonferroni correction *p* < .00029).

### 3.2 Landscape genetic analysis

The univariate layer that best described genetic patterns across the study area based on AICc was waterways smoothed at 1000 m (Table 2). The optimised resistance surface (RS) used an inverse‐reverse monomolecular transformation with a shape of 1.53 and a maximum resistance of 130.5 (Appendix S7). Resistance increased towards its maximum in areas where waterways were wider and areas where several waterways were in close proximity (i.e. <1 km apart). The binary RS for highways displayed at its original spatial scale may be considered the equal top‐performing univariate layer due to the small difference in AICc support (ΔAICc < 2). The optimised RS showed that highways acted as a facilitator of gene flow having a resistance value of 1 relative to the remainder of the study area with a resistance of 63.9 (Table 2). The binary waterways layer was the third top‐performing RS, however, because it was outperformed by waterways smoothed at 1000 m, it was not used in subsequent analysis. The FPC smoothed at 1000 m layer also outperformed distance (Table 2). This layer was optimised using a Ricker transformation with a shape of 1.77 and a maximum resistance of 56.7 (Appendix S8). The Ricker transformation maximises resistance in areas where remnant vegetation was present, but not in high density. This essentially assigns maximum resistance along the forest – crop boundary, and clustered remnant vegetation patches interrupt large continuous crop areas. No other RS outperformed the distance model, instead, multiple optimisations converged on a distance model with the binary residential areas, tracks and railways RSs all having a maximum resistance lower than 2, therefore, showing similar AICc support to the distance model (Table 2). This demonstrates an IBD trend across the landscape because every RS has an intrinsic distance effect. The model that performed the worst based on AICc support was the null model (ΔAICc = −70.3; Table 2). This model represents an island model where no landscape or geographic pattern has an influence on gene flow.

**TABLE 2:** Results of the univariate landscape genetics analysis of feral pigs (*Sus scrofa*) from far north Queensland, Australia.

Surface	AICc	ΔAICc	*R* ^2^ *m*	*R* ^2^ *c*	Transformation		*K*
Waterways smoothed at 1000 m	−526.5	0	0.554	0.736	IRM		4
Highways	−526.0	−0.5	0.456	0.732	A: 63.9	P:1	3
Waterways	−524.8	−1.7	0.499	0.726	A: 1	P:2.1	3
FPC smoothed at 1000 m	−524.5	−2.0	0.567	0.771	R		4
Distance	−524.4	−2.1	0.463	0.722	NA		2
Residential areas	−523.7	−2.8	0.463	0.720	A: 1.9	P:1	3
Tracks	−523.6	−2.9	0.468	0.723	A: 1	P:1.4	3
Railways	−523.5	−3.0	0.463	0.722	A: 1.1	P:1	3
FPC	−523.4	−3.1	0.520	0.742	IRM		4
Highways smoothed at 1000 m	−523.3	−3.2	0.448	0.748	IM		4
Slope	−522.7	−3.8	0.457	0.735	IRM		4
TWI	−522.6	−3.9	0.491	0.730	IRM		4
Elevation	−522.3	−4.2	0.463	0.723	M		4
TWI smoothed at 1000 m	−521.3	−5.2	0.412	0.715	IRM		4
Slope smoothed at 1000 m	−521.2	−5.3	0.408	0.716	IRM		4
Railways smoothed at 1000 m	−521.2	−5.3	0.409	0.715	M		4
Elevation smoothed at 1000 m	−521.2	−5.3	0.410	0.716	IRM		4
Tracks smoothed at 1000 m	−521.1	−5.4	0.408	0.714	IRM		4
Residential areas smoothed at 1000 m	−521.0	−5.5	0.409	0.716	IRM		4
Null	−456.2	−70.3	0	0.611	NA		1

*Note*: Support for single surface resistance surfaces (RSs) ranked by Akaike Information Criterion corrected for finite sample size (AICc). Marginal *R* ^2^ shows the proportion of variance explained by fixed effects and conditional *R* ^2^ shows the proportion of variance explained by both fixed and random effects. Transformation is the transformation ResistanceGA applies during optimisation. For categorical surfaces ‘A’ refers to the absence of the feature and ‘P’: refers to the presence of the feature. Transformations applied to continuous surfaces; ‘M’ denotes monomolecular, ‘IM’ denotes inverse monomolecular, ‘IRM’ denotes inverse‐reverse monomolecular and ‘R’ denotes ricker. *K* refers to the number of parameters. Grey highlight indicates surfaces at their best supported spatial scale.

**FIGURE 7:**
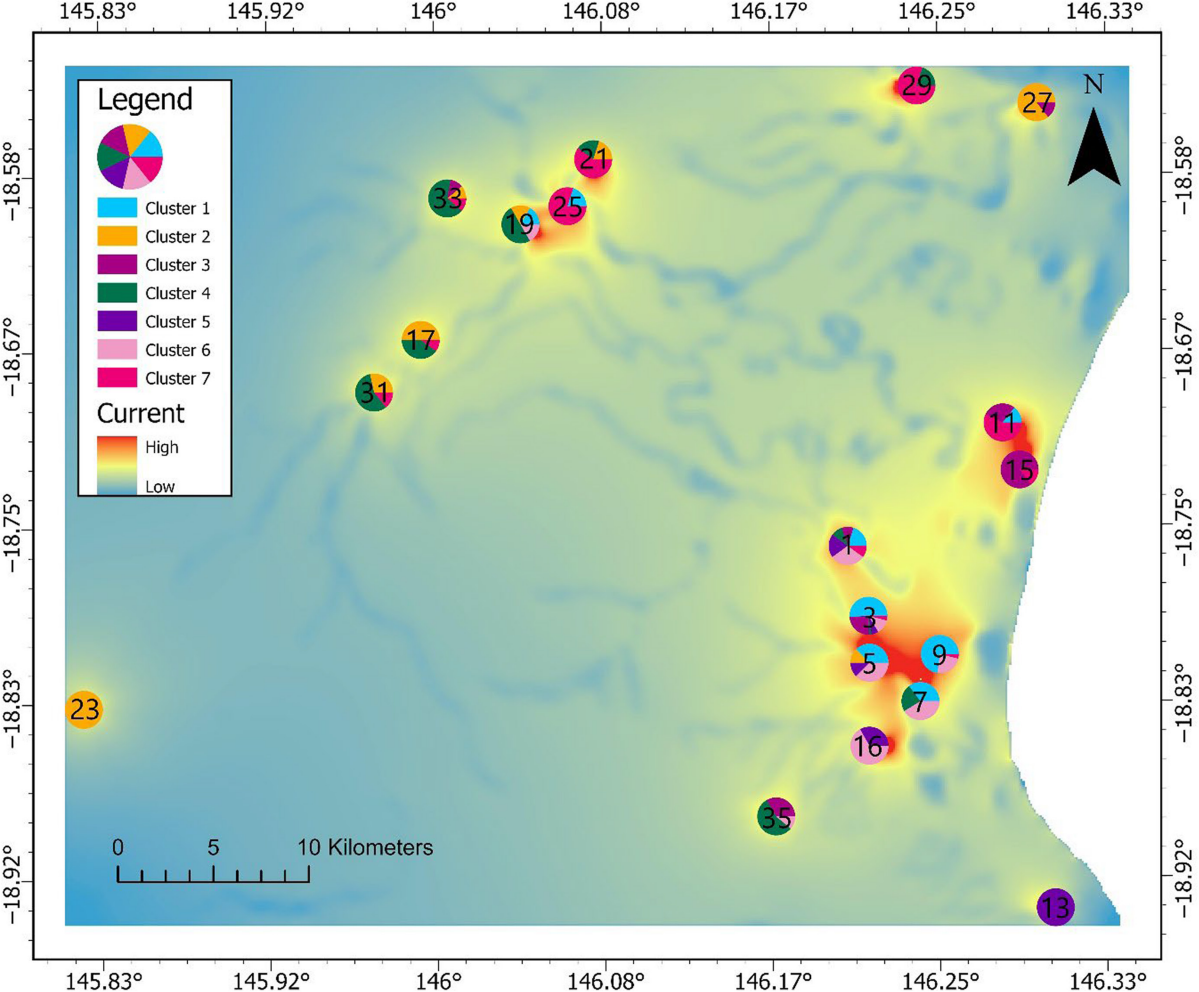
Current map in far‐north Queensland, Australia representing the current density among the 19 sites used during the ResistanceGA optimisation process for the best supported univariate resistance surface (RS; waterways smoothed at 1000 m). Higher currents indicate higher probabilities for random walkers to traverse that cell (McRae et al., 2008).

The current map (Figure 7) shows the connectivity across the landscape for the waterways smoothed at 1000 m univariate model. Connectivity between sites in the south is high, but this is most likely due to the close proximity of sites 3, 5, 7 and 9. However, the genetic distance between these sites is low (Appendix S6). There appears to be high connectivity across the Herbert River between sites 19 and 21 despite waterways being a barrier in the overall model. This may be an artefact of the close proximity of these two sites.

No combination of resistance surfaces had a correlation coefficient > 0.7 (Appendix S9), therefore, no combinations were excluded from the multivariate analysis. The multivariate analysis revealed that five models were better than the univariate distance model (Table 3). The top three multivariate layers may be considered equally well‐supported based on AICc (ΔAICc < 2). Firstly, in the combination of the highways and railways binary layers, highways had a resistance of one and, therefore, were a facilitator of gene flow, while railways had a resistance of 1481.7 indicating that they were a barrier. The absence of either feature in the RS was assigned a resistance of 232.6. Secondly, the optimised combination of waterways smoothed at 100 m and FPC smoothed at 1000 m (Table 3) retained the same transformations for each layer as was applied in the univariate analysis. This resulted in the maximum resistance values (1126.8) being assigned to the widest waterways, and high resistance (125.1) assigned to areas of low‐density remnant vegetation. Thirdly, the combination of waterways smoothed at 1000 m and highways had an optimised RS with a maximum resistance of 85.7 assigned to the widest waterways, a minimum resistance of 1 was assigned to highways, and the absence of either feature was assigned a resistance of 18.9. Four of the top five performing multivariate RSs included waterways smoothed at 1000 m. The third, fourth and fifth‐ranked RSs were all combinations including waterways smoothed at 1000 m and a binary anthropogenic landscape (Table 3). Across all three RSs, waterways were the most resistant landscape feature, railways were facilitators of gene flow with a resistance of 1, while tracks inhibited gene flow slightly with a resistance of 2. No other combination outperformed the distance model.

**TABLE 3:** Results of the multivariate landscape genetics analysis of feral pigs (*Sus* scrofa) from far‐north Queensland, Australia.

Surface	AICc	ΔAICc	*R* ^2^ *m*	*R* ^2^ *c*	*K*
Highways and railways	−526.5	0	0.538	0.761	5
Waterways smoothed at 1000 m and FPC smoothed at 1000 m	−525.7	−0.8	0.686	0.794	7
Waterways smoothed at 1000 m and highways	−524.7	−1.8	0.605	0.753	6
Waterways smoothed at 1000 m and tracks	−524.5	−2.0	0.619	0.753	6
Waterways smoothed at 1000 m and railways	−524.5	−2.0	0.614	0.754	6
Waterways smoothed at 1000 m and residential areas	−524.2	−2.3	0.613	0.753	6
Highways and tracks	−523.6	−2.9	0.459	0.745	5
Highways and residential areas	−522.6	−3.9	0.457	0.729	5
Waterways smoothed at 1000 m and Slope	−521.7	−4.8	0.620	0.777	7
FPC smoothed at 1000 m and railways	−521.6	−4.9	0.599	0.757	6
Waterways smoothed at 1000 m and TWI	−520.9	−5.6	0.630	0.762	7
FPC smoothed at 1000 m and tracks	−520.5	−6.0	0.596	0.767	6
FPC smoothed at 1000 m and slope	−520.1	−6.4	0.592	0.785	7
Highways and FPC smoothed at 1000 m	−520.0	−6.5	0.586	0.767	6
FPC smoothed at 1000 m and residential areas	−520.0	−6.5	0.572	0.761	6
Highways and slope	−519.1	−7.4	0.455	0.730	6
Waterways smoothed at 1000 m and elevation	−518.9	−7.6	0.622	0.789	7
Highways and TWI	−518.8	−7.9	0.477	0.725	6
FPC smoothed at 1000 m and elevation	−518.5	−8.0	0.586	0.782	7
FPC smoothed at 1000 m and TWI	−516.6	−9.9	0.601	0.769	7
Highways and elevation	−512.0	−14.5	0.428	0.737	6

*Note*: Support for multivariate resistance surfaces (RSs) ranked by the Akaike Information Criterion corrected for finite sample size (AICc). Each model uses pairwise linearised F_ST_ as the response variable. Grey highlight indicates models with higher AICc support than the univariate distance model. Marginal *R* ^2^ shows the proportion of variance explained by fixed effects and conditional *R* ^2^ shows the proportion of variance explained by both fixed and random effects. ‘*K*’ refers to the number of parameters.

**FIGURE 8:**
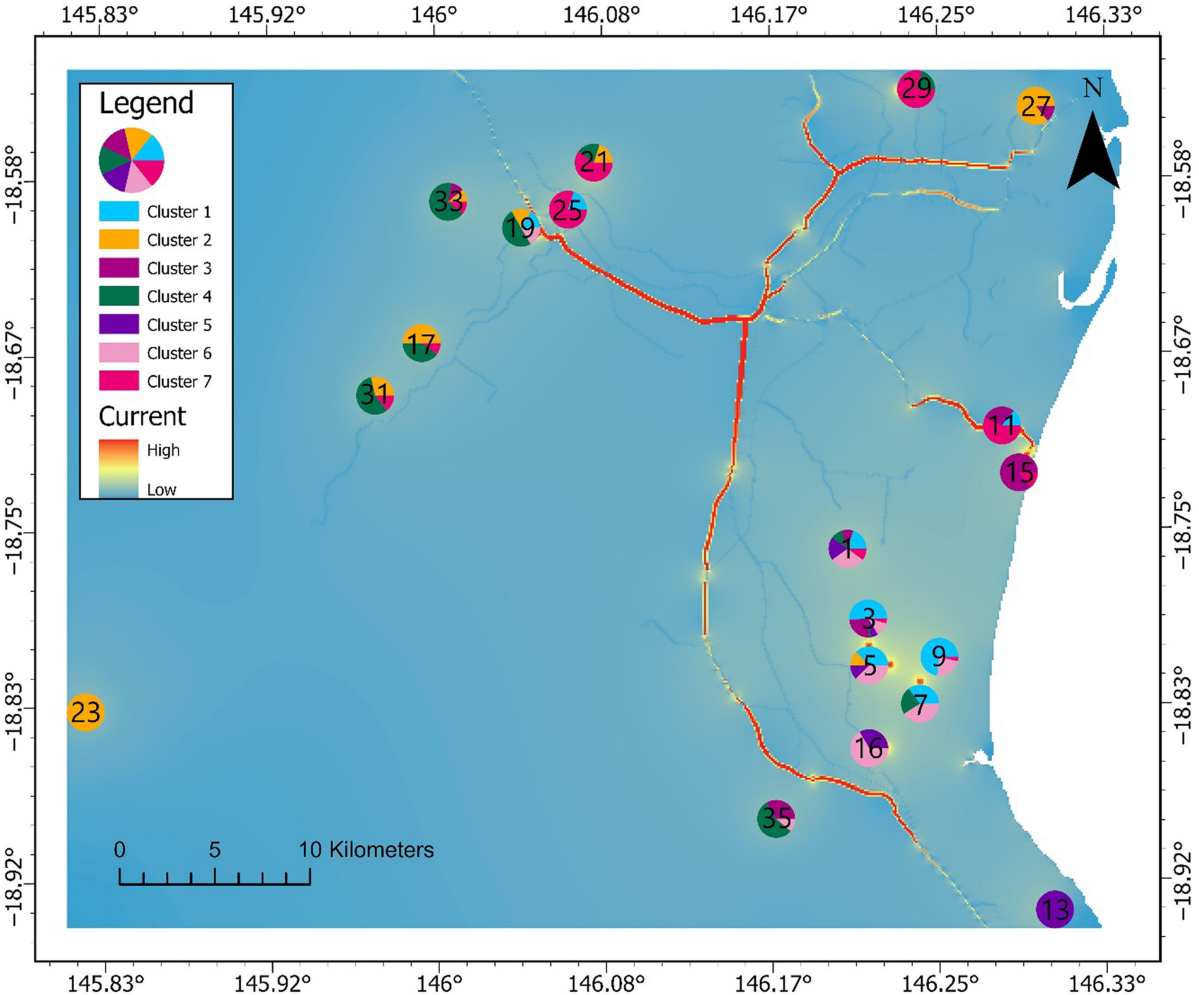
Current map in far‐north Queensland, Australia representing the current density among the 19 sites used during ResistanceGA optimisation process for the best supported multivariate resistance surface (RS; highways and railways). Higher currents indicate higher probabilities for random walkers to traverse that cell (McRae et al., 2008).

The highways and railways multivariate model shows a very high current density along the highways (Figure 8). The current along the highways appears to link several distant sites, particularly sites 11 and 15, which are two of the few sites south of the Herbert River, that exhibit cluster 5 ancestry and are also located near highways. Additionally, site 27, located near a highway, was the only site in the nornortheastth cluster 2 representation.

The bootstrap analysis revealed moderate to high Akaike weight (ω) support for four resistance surfaces. Waterways smoothed at 1000 m had the strongest support and was most robust to sub‐sampling with ω = 0.235 and was the top ranked RS for 41.75% of bootstraps (Table 4). Distance was the second ranked model (ω = 0.191) and was the top ranked model for 19.84% of bootstraps (Table 4). Distance outperformed highways and FPC smoothed at 1000 m in this bootstrap analysis because the strong support for highways and FPC smoothed at 1000 m is driven by fewer pairwise comparisons. Highways was the top ranked RS for 20.14% of bootstraps, however, the model had lower support (ω = 0.164) than distance due to its lower average rank (0.28; Table 4). Despite FPC smoothed at 1000 m having an average rank well below waterways smoothed at 1000 m, distance and highways (Table 4), it was still the top ranked RS for 16.48% of bootstraps and was the only other RS with ω > 0.1 (Table 4). Highways and railways, slope, waterways smoothed at 1000 m and tracks and FPC smoothed at 1000 m and residential areas were all top ranked resistance surfaces on at least one occasion (Table 4; Appendix S10), however, they had poor average ranks in the bootstrap analysis. This indicates that these RSs may occasionally fill niche roles in predicting genetic patterns, however, they are not affecting population structure across the overall study area.

**TABLE 4:** Results of the bootstrap landscape genetics analysis of feral pigs (*Sus scrofa*) from far‐north Queensland, Australia.

Surface	ω	Average rank	% top‐ranked	*K*
Waterways smoothed at 1000 m	0.235	3.0	41.75	4
Distance	0.191	2.3	19.84	2
Highways	0.164	2.8	20.14	3
FPC smoothed at 1000 m	0.119	6.1	16.48	4
Highways and railways	0.047	7.4	0.73	5
TWI	0.044	6.1	0	4
Slope	0.041	7.8	0.45	4
Elevation	0.035	7.2	0	4
Waterways smoothed at 1000 m and tracks	0.024	11.0	0.6	6
Highways and tracks	0.016	11.5	0	5

*Note*: Shown are the top 10 performing resistance surfaces (RSs) from the bootstrap analysis; Akaike weight (ω), average rank of the model per bootstrap, percentage of times a resistance surface (RS) was top ranked based on Akaike Information Criterion corrected for finite sample size (AICc), and number of parameters in the model (K). Full table is shown in Appendix S10.

### 3.3 Management units

We propose three management units (MUs) with boundaries that coincide with major waterways in the Herbert region because they were identified as the strongest barrier in the landscape genetics analysis. Although FPC smoothed at 1000 m performed well in the univariate analysis, it was weakly supported as a barrier to gene flow in the bootstrap analysis, and therefore remnant woody vegetation cover was not considered when defining MU boundaries. As a result, the proposed MUs are:

MU1: The western boundary coincides with the Gowrie Creek, the southern boundary is the Herbert River, the eastern boundary is the Queensland coast and the northern boundary could not be determined.
MU2: The western boundary partially coincides with two waterways; Stoney Creek and Douglas Creek, the northern boundary is the Herbert River, the eastern boundary partially coincides with two waterways; Stone River and Oaky Creek, and the southern boundary could not be determined.
MU3: The western boundary partially coincides with two waterways; Stone River and Oaky Creek, the northern boundary is the Herbert River, the southern boundary partially coincides with two waterways; Crystal Creek and Running River, and the eastern boundary is the Queensland Coast.

**FIGURE 9:**
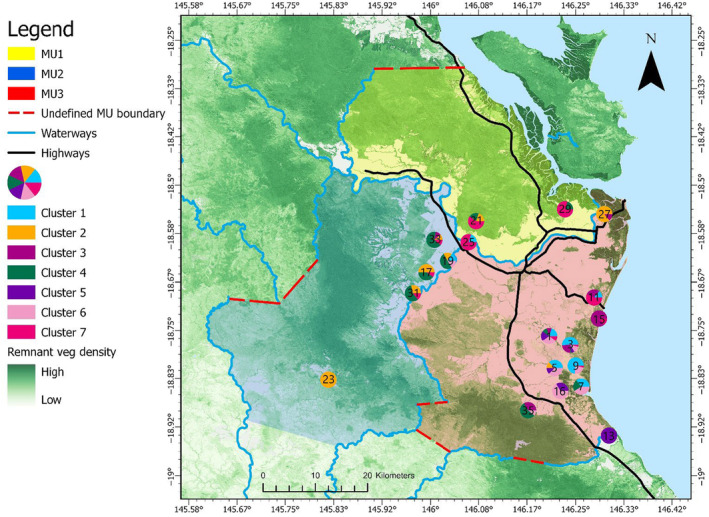
Proposed management units (MUs) of feral pigs (*Sus scrofa*) in far‐north Queensland, Australia based on results from the landscape genetics analysis. Each shaded area represents a separate MU; yellow, MU1; blue, MU2; pink, MU3. The dashed red line represents undefined MU boundaries.

No MU could be fully defined due to limits in sample coverage in locations lacking a barrier, and as such areas where boundaries remain undefined are represented as red dashed lines (Figure 9). Finally, site 13 is located South of MU3 and is possibly part of a separate MU due to its genetic distinctiveness, however, a fourth MU has not been proposed due to the lack of samples from other more southerly sites.

## 4 DISCUSSION

The primary objective of this study was to identify the landscape features that best explain the population structure of feral pigs in the Herbert region of far north Queensland. Our findings indicate an isolation by resistance (IBR) model based on major waterways was the best predictor of population structure, however isolation by distance (IBD) was also supported. These results can be used to aid feral pig management in the study area by more explicitly defining management units and population control in the study area.

### 4.1 Population and landscape structure

Major waterways were identified as the best predictor of population structure, exhibiting a minor barrier effect to gene flow in the current study. This has also been demonstrated in Portugal and Germany where major rivers were found to be barriers to wild pig gene flow (Ferriera et al., 2006, 2009; Reiner et al., 2021). In our analysis, waterways smoothed at 1000 m outperformed waterways at its original spatial scale. This indicates that wider rivers are imposing a higher resistance to feral pig gene flow than narrow rivers, however, due to the levels of admixture across the study area they appear only to be a minor barrier to gene flow. Feral pigs in Australia have previously been shown to move along waterways in low average annual rainfall (300–500 mm) environments (Cowled et al., 2008; Hampton et al., 2004), however, they are less likely to use waterways as movement pathways in high average annual rainfall (500–700 mm) environments (Cowled et al., 2008). Given the current study was undertaken in an extreme rainfall environment (annual average ≥ 2000 mm), our findings reinforce the notion that waterways are not movement pathways in high rainfall environments, but instead act as major barriers to gene flow. The study areas of both Cowled et al. (2008) and Hampton et al. (2004) were environments with lower average annual rainfall than the current study, and with limited food and water availability, thus restricting feral pigs to permanent water sources. Far north Queensland is a resource‐abundant environment (Mitchell et al., 2009) which does not restrict feral pigs to permanent water sources. Additionally, feral pigs are likely to display anti‐predatory behaviours in a resource‐rich environment (Clark, 1994; Morris et al., 2009), which may deter them from crossing waterways due to the risk of saltwater crocodile (*Crocodylus porosus*) predation (Bowman & McDonough, 1991; Gruen, 2009; Mitchell, 2010).

Our study found a significant pattern of IBD among sampling sites. Isolation by distance has often been observed in other wild pig population genetics analyses (Choi et al., 2014; Cowled et al., 2008; Frantz et al., 2009, 2012; Renner et al., 2016; Rutten et al., 2019). The current data and that of others who observed IBD indicate that limited movement has an important influence on genetic structure in wild pig populations. An IBD pattern is unsurprising in our study area due to the generally sedentary nature of feral pigs in far‐north Queensland (Caley, 1997; Lopez et al., 2014; Mitchell et al., 2009). However, distance does not fully explain the population genetic patterns of feral pigs in far north Queensland. Distance was outperformed by waterways (both spatial scales), FPC smoothed at 1000 m and highways in the univariate analysis, however, it was only outperformed by waterways smoothed at 1000 m in the bootstrap analysis, indicating that few key sites had a strong influence on the RS optimisation of highways and FPC smoothed at 1000 m. Due to the possibility of including related individuals in our analysis, it is possible that this may have biased our results towards a pattern of IBD. However, because IBD is commonly found in wild pig populations, and has previously been identified in Australian feral pig populations (Cowled et al., 2008), it is plausible that our observed IBD pattern is not the result of a type 1 error.

In our study, highways appear to facilitate feral pig gene flow in far north Queensland. This result could be due to two processes: (1) long‐distance dispersal where highways act as corridors or (2) translocation events. Previous research indicates that feral pigs in tropical Australian habitats are generally sedentary (Caley, 1997; Lopez et al., 2014; Mitchell et al., 2009) with an average movement distance of 1 km from the centre of their home ranges (Mitchell et al., 2009). A long term (1989–1992) mark and recapture study investigating dispersal of feral pigs in the Northern Territory, Australia, found the mean recapture distance of male pigs to be 3.2 km and for females 1.8 km (Caley, 1997), which is consistent with other literature demonstrating that feral pigs in Australia, and particular tropical habitats, are sedentary. Additionally, Australian studies have detected evidence of feral pig translocation events (Hampton et al., 2004; Spencer & Hampton, 2005). Our results are not conclusive; therefore, unassisted long‐distance dispersal along highways, translocation or a combination of both processes is a possibility. Our result also appears to be strongly influenced by specific sites. For example, site 35 is located nearby to a highway and is highly admixed (low pairwise F_ST_ values). Feral pigs found nearby to public vehicle access have previously been identified as possible translocations in Australian studies (Spencer & Hampton, 2005). Other studies on wild pigs found that highways exhibited no influence on population genetic structure (Dellicour et al., 2019; Frantz et al., 2012), however, Dellicour et al. (2019) proposed that not enough time had passed since the highways were constructed for genetic divergence to become detectable.

### 4.2 Management implications

We propose that three MUs exist in the study area. Management conducted across an area smaller than the extent of a feral pig population has previously been shown to be ineffectual due to reinvasion (Cowled et al., 2008). Therefore, management conducted on a smaller scale than the three MUs proposed in this study (e.g. control at a property/farm level or ad hoc areas based on governmental jurisdiction) or non‐simultaneously within a MU is unlikely to be effective in reducing feral pig population numbers long term. Due to the absence of observed genetic discontinuities at the extremities of the study area, we cannot define MUs based on the definition provided by Moritz (1994), instead MU boundaries have been delineated based on key landscape features identified in our analysis. Consequently, MU boundaries were primarily defined according to the presence of major waterways in the study area, which we have found to act as a minor barrier to gene flow, and may aid in minimising reinvasion potential and hinder population growth within a MU.

Only weak support was found for remnant vegetation edges acting as a barrier to gene flow, and consequently, we did not define any MU boundaries based on the presence of remnant vegetation. However, we endorse that previous management recommendations for simultaneous management of feral pigs in cropland habitats and adjacent rainforest habitats (Lopez et al., 2014; Mitchell et al., 2009) should be considered when designing control programs. While we have focussed on defining MU boundaries based on natural barriers to gene flow, it should be noted that these will not prevent the movement of feral pigs along highways (which were found to facilitate gene flow in this study) or recolonisation from possible human‐mediated translocation of feral pigs among MUs (Smith et al., 2005). Previous recommendations aimed at preventing human‐mediated translocations include law enforcement and education (Cowled et al., 2009) and it is recommended that the same strategies should be employed in the Herbert region of far‐north Queensland.

The boundaries of each of the three MUs proposed in the current study cannot be completely defined because the genetic relationships with populations beyond the study area remain unknown. It is possible that the population boundaries extend further than the MUs proposed in this study, and in fact, due to the IBD pattern identified it is not unreasonable to assume that this pattern extends beyond our study area. Additionally, anecdotal observations indicate that feral pigs do traverse the Herbert River when water levels are low (Di Bella, personal communication). This specifically has been observed between the Lannercost (site 19) and Hawkins Creek (site 21). Therefore, feral pig management plans developed for the Herbert region should include subsequent monitoring to determine the efficacy of current MU boundaries on minimising reinvasion potential. Additionally, due to our inability to identify population boundaries at the edges of our study, it is possible that MUs extend beyond the areas presented in this study.

### 4.3 Assumptions, limitations and future directions

There are several assumptions and limitations of the current study. The computational intensity of the genetic algorithm implemented in ResistanceGA (Peterman, 2018) prevented finer spatial scales from being included in the landscape genetic analysis. Binary RS were converted from vector data into raster data at a cell size of 100 m × 100 m, and finer scale continuous layers were coarsened, potentially sacrificing some ecological information. However, layer coarsening has been shown to have minimal impacts in circuit‐theory‐based approaches (Cushman & Landguth, 2010; McRae et al., 2008), and because two of the top‐performing models were derived from a 1000 m Gaussian kernel smoothing, the coarsening of vector layers into 100 m × 100 m raster layers likely did not affect the analysis (Winiarski et al., 2020). In addition, intermediate bandwidths in kernel smoothing were not assessed due to time constraints, however, 1000 m was considered to be the optimal secondary spatial scale given existing literature on feral pig movement patterns in far‐north Queensland (Mitchell et al., 2009). Previous research has demonstrated that the artificial boundaries of RSs can bias current densities at the edges of the RS upwards (Koen et al., 2010). It is possible that the 1 km buffer applied to our study area was inadequate, however, because the current maps we generated (Figures 6 and 7) do not display an increase in current density at RS boundaries, we conclude that a 1 km buffer was sufficient.

The effect of FPC cannot be fully assessed due to sampling design. Only site 23 was separated from all other sites by a large area of remnant woody vegetation, and all other sites were sampled in low‐lying areas of cropland or on the crop‐forest interface. This means that the analysis lacks statistical power to determine if there is a barrier effect on feral pig gene flow between cropland and rainforest habitats. Linearised pairwise F_ST_ values for comparisons including site 23 indicate significant genetic differentiation between this site and all others. However, the PCA did not reveal any notable partitioning of site 23. Due to this limitation, the optimised FPC RS in the current study was a function of its influence on the gene flow of feral pigs in cropland habitats and may not reflect its true influence (if any) on feral pig population structure.

Future research in far‐north Queensland should aim to sample from both high‐elevation rainforest sites and low‐lying agricultural areas. It is also possible that non‐random mating among pig breeds may have an influence on the genetic structure as this has previously been hypothesised in a study undertaken in far‐north Queensland (Lopez et al., 2014). Finally, key landscape features identified in this study may not be good predictors of feral pig population structure in other regions of Australia, particularly as waterways are movement pathways in low rainfall environments (Cowled et al., 2008; Hampton et al., 2004). Therefore, additional landscape genetic analyses should be conducted to inform feral pig management strategies in different ecosystems across Australia.

## AUTHOR CONTRIBUTIONS

**James Ryan:** Data curation (lead); formal analysis (lead); writing – original draft (lead); writing – review and editing (equal). **Peter J. Prentis:** Formal analysis (supporting); supervision (equal); writing – original draft (supporting); writing – review and editing (equal). **Susan Fuller:** Conceptualization (lead); formal analysis (supporting); supervision (equal); writing – original draft (supporting); writing – review and editing (equal).

## CONFLICT OF INTEREST STATEMENT

The authors declare no competing interests.

### OPEN RESEARCH BADGES

This article has earned Open Data, Open Materials and Preregistered Research Design badges. Data, materials and the preregistered design and analysis plan are available at https://osf.io/5xuak/.

## Supporting information


Appendix S1.

## Data Availability

Microsatellite data and raster layers: Open Science Framework https://osf.io/5xuak/.
