# Supplementary material for: Multiscale landscape genetic analysis identifies major waterways as a barrier to dispersal of feral pigs in north Queensland, Australia
Source: Ecol Evol. 2023 Sep 28;13(10):e10575. doi: 10.1002/ece3.10575 (PMC10539047; doi:10.1002/ece3.10575)
Supplement: Supplementary file 1 — Appendix S1. [file ECE3-13-e10575-s001.docx]

**Appendices:**

Appendix 1 - Layer information including the name of the spatial layer retrieved from Qspatial, the corresponding resistance surface, and the type of resistance surface developed for our landscape genetics analysis of feral pigs (*Sus scrofa*) in far-north Queensland, Australia. In this study the term “tracks” has been used to describe publicly assessable roadways which are not highways or main roads.

| Layer name (Source) | Resistance surface | Type |
| --- | --- | --- |
| Baseline roads and tracks (Qspatial) | Highways | Categorical |
| Baseline roads and tracks (Qspatial) | Tracks | Categorical |
| Rail network (Qspatial) | Railways | Categorical |
| Watercourse areas, major watercourse lines & Land use mapping – 1999 to current (Qspatial) | Waterways | Categorical |
| Land use mapping – 1999 to current (Qspatial) | Residential areas | Categorical |
| Topographic wetness index (CSIRO) | Topographic wetness index | Continuous |
| Digital elevation model – 25m – Burdekin catchment & Digital elevation model – 25m – Wet Tropics (Qspatial) | Elevation | Continuous |
| Digital elevation model – 25m – Burdekin catchment & Digital elevation model – 25m – Wet Tropics (Qspatial) | Slope | Continuous |
| Wooded extent and foliage projective cover code layer – Queensland 2012 (Qspatial) | Remnant Vegetation | Continuous |


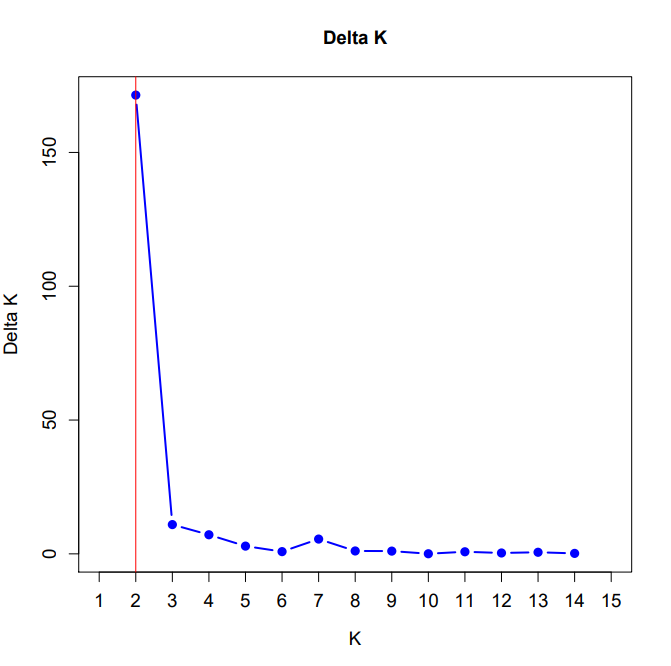


Appendix 2 – ∆*K* support for *K* values from 2 – 15 according to the Evanno method implemented in the online software StructureSelector.


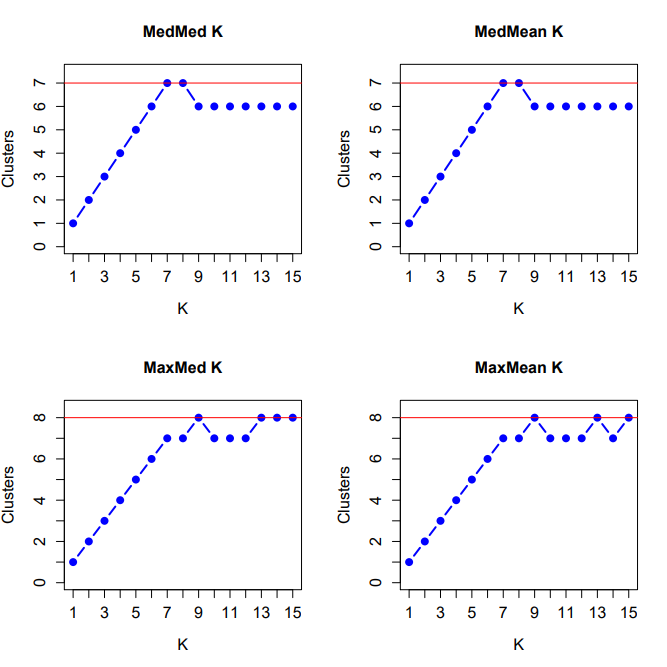


Appendix 3 – Support for values for *K* from 1 – 15 according to the Puechmaille method as implemented in the online software StructureSelector for each of the four metrics: median of medians (MedMedK*)*, the median of means (MedMeanK), the maximum of medians (MaxMedK) and the maximum of means (MaxMeanK).


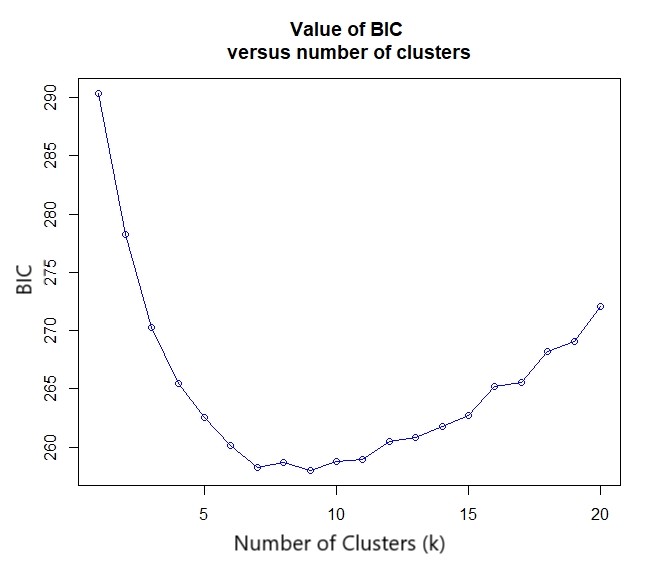


Appendix 4 – Bayesian Information Criterion (BIC) values for values of *K* from 1 – 20 determined using the *find.clusters()* function in the adegenet R package. According to BIC the optimal value *is* *K* = 7.


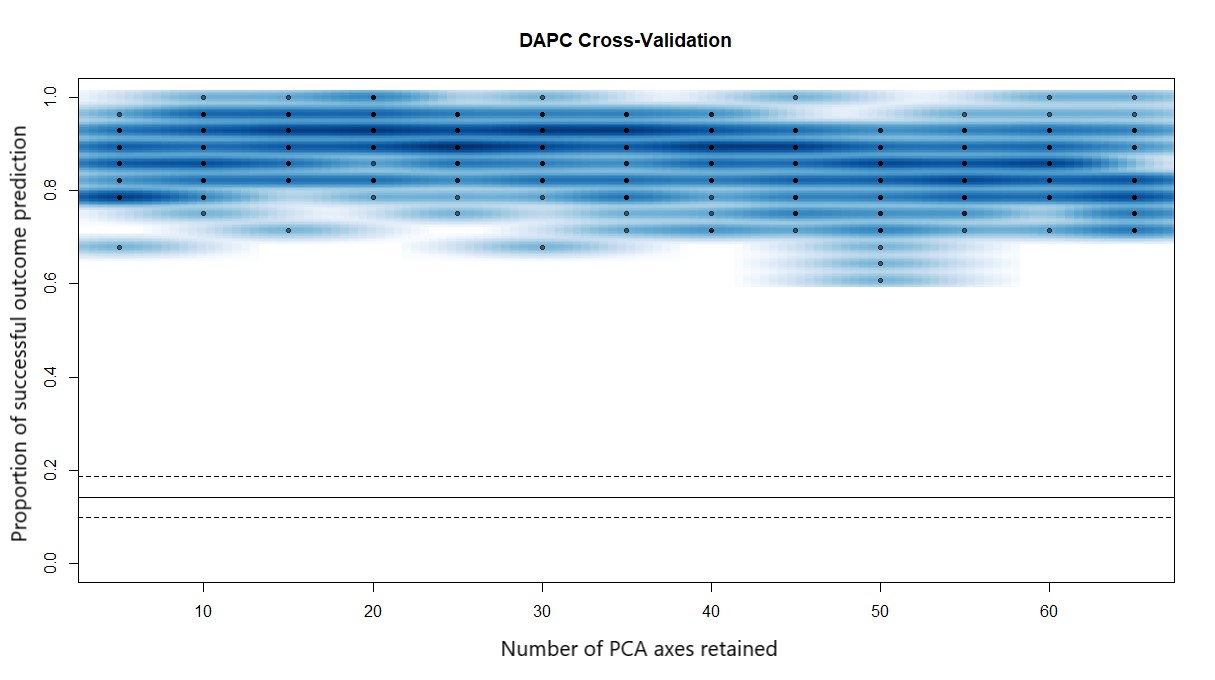


Appendix 5 – Discriminant analysis of principal components (DAPC) cross validation success as a proportion of successful prediction outcomes where each point is a replicate for each number tested principal components (PCs).

Appendix 6 – Lower pairwise linearised F_ST_ matrix of feral pigs (*Sus scrofa*) collected from far-north Queensland, Australia. Red text denotes non-significance after Bonferroni correction (p < 0.00029)

| Site | 1 | 3 | 5 | 7 | 9 | 11 | 13 | 15 | 16 | 17 | 19 | 21 | 23 | 25 | 27 | 29 | 31 | 33 | 35 |
| --- | --- | --- | --- | --- | --- | --- | --- | --- | --- | --- | --- | --- | --- | --- | --- | --- | --- | --- | --- |
| 1 | * |  |  |  |  |  |  |  |  |  |  |  |  |  |  |  |  |  |  |
| 3 | 0.037 | * |  |  |  |  |  |  |  |  |  |  |  |  |  |  |  |  |  |
| 5 | 0.047 | 0.014 | * |  |  |  |  |  |  |  |  |  |  |  |  |  |  |  |  |
| 7 | 0.063 | 0.016 | 0.005 | * |  |  |  |  |  |  |  |  |  |  |  |  |  |  |  |
| 9 | 0.081 | 0.022 | 0.050 | 0.038 | * |  |  |  |  |  |  |  |  |  |  |  |  |  |  |
| 11 | 0.051 | 0.046 | 0.072 | 0.071 | 0.107 | * |  |  |  |  |  |  |  |  |  |  |  |  |  |
| 13 | 0.222 | 0.232 | 0.235 | 0.248 | 0.372 | 0.200 | * |  |  |  |  |  |  |  |  |  |  |  |  |
| 15 | 0.127 | 0.114 | 0.098 | 0.162 | 0.199 | 0.107 | 0.387 | * |  |  |  |  |  |  |  |  |  |  |  |
| 16 | 0.154 | 0.139 | 0.077 | 0.147 | 0.215 | 0.152 | 0.255 | 0.241 | * |  |  |  |  |  |  |  |  |  |  |
| 17 | 0.180 | 0.167 | 0.176 | 0.140 | 0.220 | 0.219 | 0.495 | 0.251 | 0.309 | * |  |  |  |  |  |  |  |  |  |
| 19 | 0.108 | 0.069 | 0.081 | 0.069 | 0.113 | 0.089 | 0.284 | 0.123 | 0.205 | 0.084 | * |  |  |  |  |  |  |  |  |
| 21 | 0.073 | 0.112 | 0.085 | 0.096 | 0.155 | 0.102 | 0.378 | 0.083 | 0.215 | 0.023 | 0.042 | * |  |  |  |  |  |  |  |
| 23 | 0.153 | 0.167 | 0.118 | 0.159 | 0.225 | 0.190 | 0.336 | 0.175 | 0.123 | 0.220 | 0.164 | 0.103 | * |  |  |  |  |  |  |
| 25 | 0.181 | 0.178 | 0.200 | 0.163 | 0.247 | 0.084 | 0.522 | 0.199 | 0.378 | 0.242 | 0.116 | 0.022 | 0.219 | * |  |  |  |  |  |
| 27 | 0.231 | 0.175 | 0.177 | 0.246 | 0.243 | 0.250 | 0.481 | 0.201 | 0.269 | 0.157 | 0.182 | 0.100 | 0.194 | 0.348 | * |  |  |  |  |
| 29 | 0.172 | 0.169 | 0.157 | 0.182 | 0.229 | 0.169 | 0.606 | 0.227 | 0.253 | 0.301 | 0.194 | 0.106 | 0.240 | 0.187 | 0.308 | * |  |  |  |
| 31 | 0.135 | 0.116 | 0.110 | 0.099 | 0.181 | 0.129 | 0.409 | 0.159 | 0.172 | 0.034 | 0.051 | 0.020 | 0.133 | 0.233 | 0.169 | 0.178 | * |  |  |
| 33 | 0.100 | 0.083 | 0.067 | 0.081 | 0.146 | 0.086 | 0.379 | 0.112 | 0.165 | 0.058 | 0.043 | 0.036 | 0.162 | 0.150 | 0.175 | 0.146 | 0.000 | * |  |
| 35 | 0.040 | 0.027 | 0.000 | 0.020 | 0.046 | 0.082 | 0.275 | 0.122 | 0.089 | 0.129 | 0.061 | 0.058 | 0.144 | 0.212 | 0.181 | 0.139 | 0.095 | 0.074 | * |


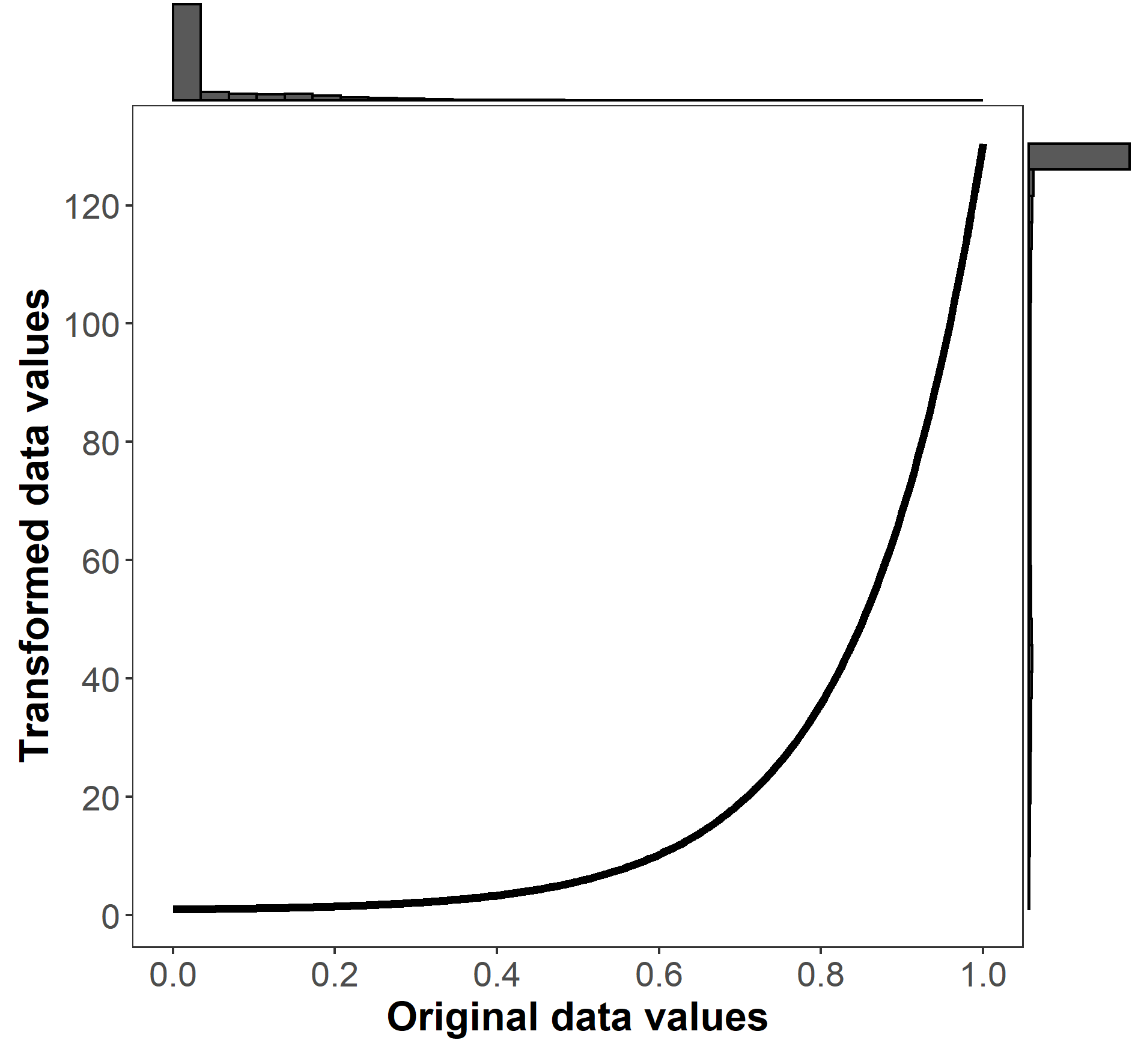


Appendix 7 – Transformation applied to waterways smoothed at 1000m during the ResistanceGA landscape genetics optimisation process.


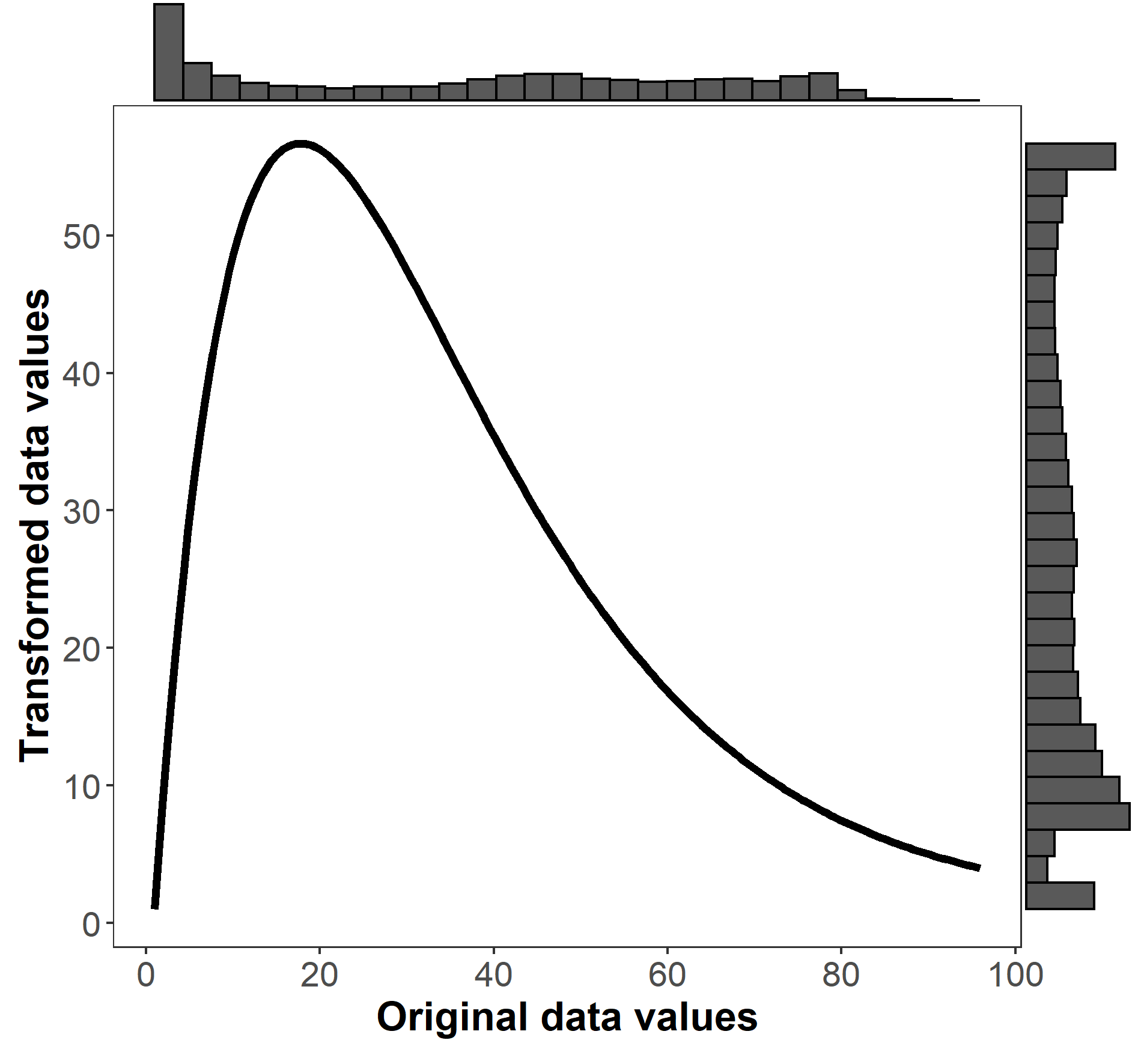


Appendix 8 – Transformation applied to Foliage Projective Cover (FPC) smoothed at 1000m during the ResistanceGA landscape genetics optimisation process.

Appendix 9 – Pearson’s correlation coefficient for each combination of resistance surface (RS) analysed in the multivariate landscape analysis of feral pigs (*Sus scrofa*) in far-north Queensland, Australia. Included is Foliage Projective Cover (FPC) and Topographic Wetness Index (TWI).

| Layer Combination | Waterways | Highways | FPC | Elevation | Railways | Residential Areas | Slope | Tracks | TWI |
| --- | --- | --- | --- | --- | --- | --- | --- | --- | --- |
| Waterways | NA | 0.0471 | -0.2757 | -0.3302 | 0.0691 | 0.0593 | -0.2740 | 0.0331 | 0.2773 |
| Highways | 0.0471 | NA | -0.0817 | -0.0623 | 0.1979 | 0.1460 | -0.0515 | 0.0368 | 0.0476 |
| FPC | -0.2757 | -0.0817 | NA | 0.5960 | -0.1475 | -0.0926 | 0.5306 | -0.1040 | -0.5630 |

Appendix 10 – Full results of the bootstrap landscape genetics analysis of feral pigs (*Sus scrofa*) from far-north Queensland, Australia. Models are ranked according to Akaike weight (ω). Also reported is the average rank of the model per bootstrap, percentage of times a resistance surface (RS) was top ranked based on Akaike Information Criterion corrected for finite sample size (AICc), and number of parameters in the model (K). Included are Foliage Projective Cover (FPC) and Topographic Wetness Index (TWI).

| Surface | ω | Average Rank | Top Ranked | % Top Ranked | K |
| --- | --- | --- | --- | --- | --- |
| Waterways smoothed at 1000m | 0.235 | 2.962 | 4175 | 41.75 | 4 |
| Distance | 0.191 | 2.269 | 1984 | 19.84 | 2 |
| Highways | 0.164 | 2.843 | 2014 | 20.14 | 3 |
| FPC smoothed at 1000m | 0.119 | 6.115 | 1648 | 16.48 | 4 |
| Highways & Railways | 0.047 | 7.366 | 73 | 0.73 | 5 |
| TWI | 0.044 | 6.111 | 0 | 0 | 4 |
| Slope | 0.041 | 7.821 | 45 | 0.45 | 4 |
| Elevation | 0.035 | 7.152 | 0 | 0 | 4 |
| Waterways smoothed at 1000m & Tracks | 0.024 | 11.013 | 60 | 0.6 | 6 |
| Highways & Tracks | 0.016 | 11.486 | 0 | 0 | 5 |
| Waterways smoothed at 1000m & Railways | 0.015 | 11.553 | 0 | 0 | 6 |
| Waterways smoothed at 1000m & Residential Areas | 0.015 | 12.190 | 0 | 0 | 6 |
| Waterways smoothed at 1000m & Highways | 0.014 | 11.982 | 0 | 0 | 6 |
| Highways & Residential Areas | 0.012 | 11.623 | 0 | 0 | 5 |
| FPC smoothed at 1000m & Railways | 0.008 | 14.418 | 0 | 0 | 6 |
| FPC smoothed at 1000m & Tracks | 0.005 | 16.721 | 0 | 0 | 6 |
| Highways & FPC smoothed at 1000m | 0.004 | 17.726 | 0 | 0 | 6 |
| FPC smoothed at 1000m & Residential Areas | 0.004 | 17.605 | 1 | 0.01 | 6 |
| Waterways smoothed at 1000m & FPC smoothed at 1000m | 0.002 | 19.555 | 0 | 0 | 7 |
| Highways &TWI | 0.001 | 18.658 | 0 | 0 | 6 |
| Highways & Slope | 0.001 | 19.246 | 0 | 0 | 6 |
| Waterways & TWI | 0.001 | 22.052 | 0 | 0 | 7 |
| Waterways smoothed at 1000m & Slope | 0.001 | 22.492 | 0 | 0 | 7 |
| Highways & Elevation | 0.000 | 23.836 | 0 | 0 | 6 |
| FPC smoothed at 1000m & Slope | 0.000 | 24.779 | 0 | 0 | 7 |
| FPC smoothed at 1000m & Slope | 0.000 | 24.779 | 0 | 0 | 7 |
| Waterways smoothed at 1000m & Elevation | 0.000 | 24.870 | 0 | 0 | 7 |
| FPC smoothed at 1000m & TWI | 0.000 | 25.636 | 0 | 0 | 7 |
| FPC smoothed at 1000m & Elevation | 0.000 | 25.922 | 0 | 0 | 7 |
